# Development of Single-Channel Dual-Element Custom-Made Ultrasound Scanner with Miniature Optical Position Tracker for Freehand Imaging

**DOI:** 10.3390/bios13040431

**Published:** 2023-03-28

**Authors:** Yen-Lung Chen, Huihua Kenny Chiang

**Affiliations:** Department of BioMedical Engineering, National Yang Ming Chiao Tung University, Taipei 112, Taiwan; violet83071212@gmail.com

**Keywords:** ultrasound, rectus femoris muscle, dual-element ultrasound transducer, optical tracker, custom-made ultrasound transducer, ultrasound scanner, freehand imaging

## Abstract

Handheld ultrasound has great potential in resource-limited areas, and can improve healthcare for rural populations. Single-channel ultrasound has been widely used in many clinical ultrasound applications, and optical tracking is considered accurate and reliable. In this study, we developed a 10 MHz lead magnesium niobate–lead titanate (PMN-PT) dual-element ultrasound transducer combined with a miniature optical position tracker, and then measured the rectus femoris of the thigh, upper arm, and cheek muscles. Compared to single-element transducers, dual-element transducers improve the contrast of near-field signals, effectively reduce noise, and are suitable for measuring curved surfaces. The purpose of position tracking is to calculate the location of the ultrasound transducer during the measurement process. By utilizing positioning information, 2D ultrasound imaging can be achieved while maintaining structural integrity. The dual-element ultrasound scanner presented in this study can enable continuous scanning over a large area without a scanning width limitation. The custom-made dual-element ultrasound scanner has the advantage of being a portable, reliable, and low-cost ultrasound device, and is helpful in popularizing medical care for remote villages.

## 1. Introduction

Ultrasonography has been widely used for noninvasive medical imaging. Ultrasound is suitable for the imaging of various organs, including the breast [1], abdomen [2], cardiovascular [3], gynecology [4], and skeletal muscle [5]. In these applications, multichannel array transducers have been widely used because of their advantages, including a broad field of view, superior lateral resolution, multiple zone focus and dynamic focus, compounding imaging, and real-time imaging. However, the multichannel array transducer has a complex structure, is large in size, and expensive. For specific applications, there are many clinical requirements for single-channel transducers.

Single-channel ultrasound has been widely used in many clinical applications, including ocular measurement [6], intravascular measurement [7], sinus measurement [8], blood flow measurement [9], intra-needle ultrasound application [10], and skin ultrasound measurements [11]. Garcia et al. demonstrated that intravascular ultrasound (IVUS) is an important diagnostic tool for the clinical assessment of coronary artery disease [7]. Rohr et al. used a 3.5 MHz low-frequency single-element transducer to diagnose normal or pathological sinus conditions [8]. Qiu et al. used a 40 MHz lead magnesium niobate–lead titanate (PMN-PT) single-element needle transducer to monitor the blood flow velocity near the site of a rabbit ear vein occlusion to determine the effect of sonothrombolysis [9]. Furthermore, Bok et al. demonstrated that a high-frequency (44 MHz) needle ultrasound can be used to mechanically scan images of the rabbit eye [10]. Vogt et al. demonstrated the use of a high-frequency single-element transducer (20 MHz) for high-resolution skin imaging [11].

In recent years, developments in electronic technology have led to the progressive miniaturization of handheld ultrasound systems. Tiouririne et al. developed real-time volumetric imaging of the spine using a portable ultrasound single-element (5 MHz) handheld device [12]. Choi et al. developed a single-element handheld system, and successfully measured human radial artery and vein images [13]. Falkowski et al. compared the effectiveness of portable handheld array ultrasonics to traditional cart-based ultrasonics in evaluating the musculoskeletal system; both systems exhibited high consistency [14]. Another study demonstrated that a handheld US/PA probe enables flexibility in imaging different body parts using the same probe [15]. Handheld ultrasound has great potential for resource-limited areas, can improve healthcare for rural populations, and has the unique opportunity to expand its medical applications to remote areas.

Several positioning methods have been developed for ultrasound transducers, such as the electromagnetic tracking method [16,17], electro-mechanism method [18], and robotic arm method [19,20]. Daoud et al. developed a handheld 3D ultrasound imaging system based on traditional 2D ultrasound imaging systems paired with electromagnetic tracking technology to measure and reconstruct muscle volume [16]. Lang et al. used an unscented Kalman filter to combine electromagnetic and scatter-based tracking information, thereby reducing drift errors and high-frequency noise in electromagnetic tracking to obtain accurate 3D images [17]. Lee et al. utilized a combination of 2D B-mode PA/US imaging and a mechanical scanning stage to produce high-resolution 3D images of various body parts including the neck, wrist, thigh, and instep in humans [18]. Huang et al. used a depth camera to capture point clouds of the skin surface, to determine the scan range and scan path based on the 3D contour, and then to allow a robotic arm to automatically complete the scanning, thus realizing 3D US reconstruction for the entire system [19]. Swerdlow et al. demonstrated that mechanical arms can assist ultrasound physicians in reducing stress injury and have potential applicability in robotic surgery and interventional procedures [20]. In addition, our team previously used a single-element ultrasound probe combined with an optical positioning system to scan and observe SMAS layer images of superficial skin [21].

Recently, several studies employed array-based ultrasound combined with an optical positioning tracker to realize 3D ultrasound imaging. Blackall et al. employed positional sensors to track a conventional ultrasound probe and combined 2D optical positioning tracker information with B-mode images to create volume images. The accuracy was confirmed through validation using a gel phantom model, demonstrating that it is a fast and precise handheld 3D ultrasound system [22]. Fry et al. used an optical positioning tracker for 3D ultrasound imaging reconstruction [23]. Sato et al. used a 3D optical positioning tracker to precisely determine the position and orientation of ultrasound cross-sections during breast surgeries. These data were then used to create a highly accurate 3D tumor model, which was superimposed on real-time video images of the patient’s breast, providing the surgeon with a clear 3D view of the tumor’s location [24].

In recent years, ultrasonography has become a widely used method for detecting muscle. Leahy et al. used GE 12 MHz linear array ultrasonography to measure the thicknesses of subcutaneous fat tissues in five body regions. They accurately predicted the body fat percentages of young men and women using quantile regression prediction equations [25]. Hida et al. used 5–18 MHz linear array transducer ultrasonography to determine the thickness of the thigh muscle and predict the appendicular lean soft tissue mass based on gender [26]. Abe et al. utilized 7.5 MHz linear array transducer ultrasonography similarly, measuring the thickness of the anterior forearm muscle and predicting the appendicular lean soft tissue mass based on height [27]. Souza et al. used 6–12 MHz linear array transducer ultrasonography to diagnose sarcopenia by measuring cross-sectional areas of the rectus femoris [28]. Strasser et al. used 7.25 MHz linear array transducer ultrasonography to measure the muscle pennation angle, which can only be observed through longitudinal scanning, to evaluate muscle strength [29]. As countries face an aging population, sarcopenia has become increasingly prevalent in recent years, leading to a growing health need for simple and low-cost, point-of-care ultrasound for measuring muscle thickness and cross-sectional area imaging.

In this study, we developed a single-channel, dual-element ultrasound transducer that combines with an optical position tracker to enable handheld imaging of human muscles and shallow facial tissue. The customized dual-element transducer improves the resolution of near-field signals, effectively reducing noise, and is more suitable for surface measurements due to the angle between the two chips. We used a compact optical position tracker to provide real-time displacement and enabled handheld scans without length limitations. This approach eliminates the inconvenience of the current array ultrasound transducer, which requires multiple image acquisitions and recombination for the acquisition of a large area, as well as the scanning limitations imposed by electromechanical and mechanical arm systems due to the length of the equipment.

## 2. Materials and Methods

### 2.1. Dual-Element Ultrasound Transducer

**Figure 1:**
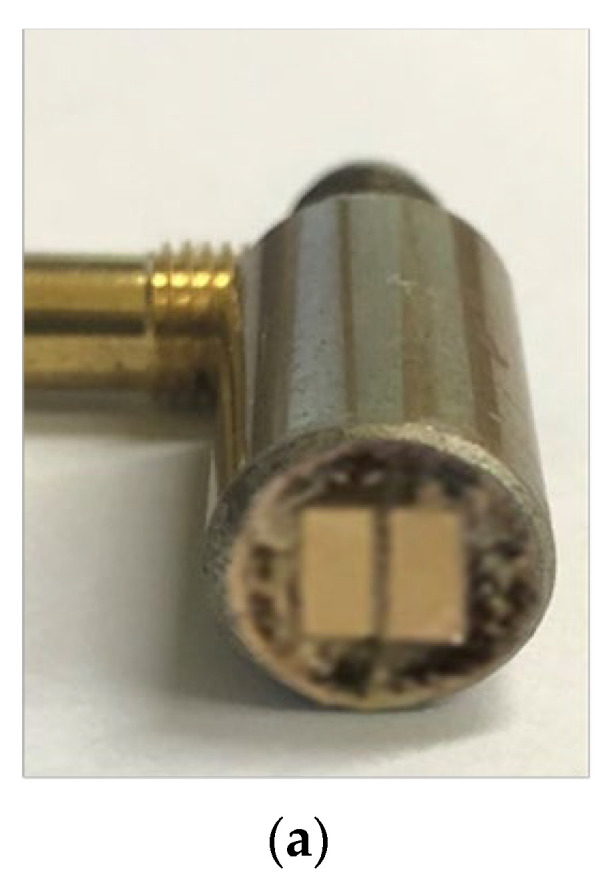

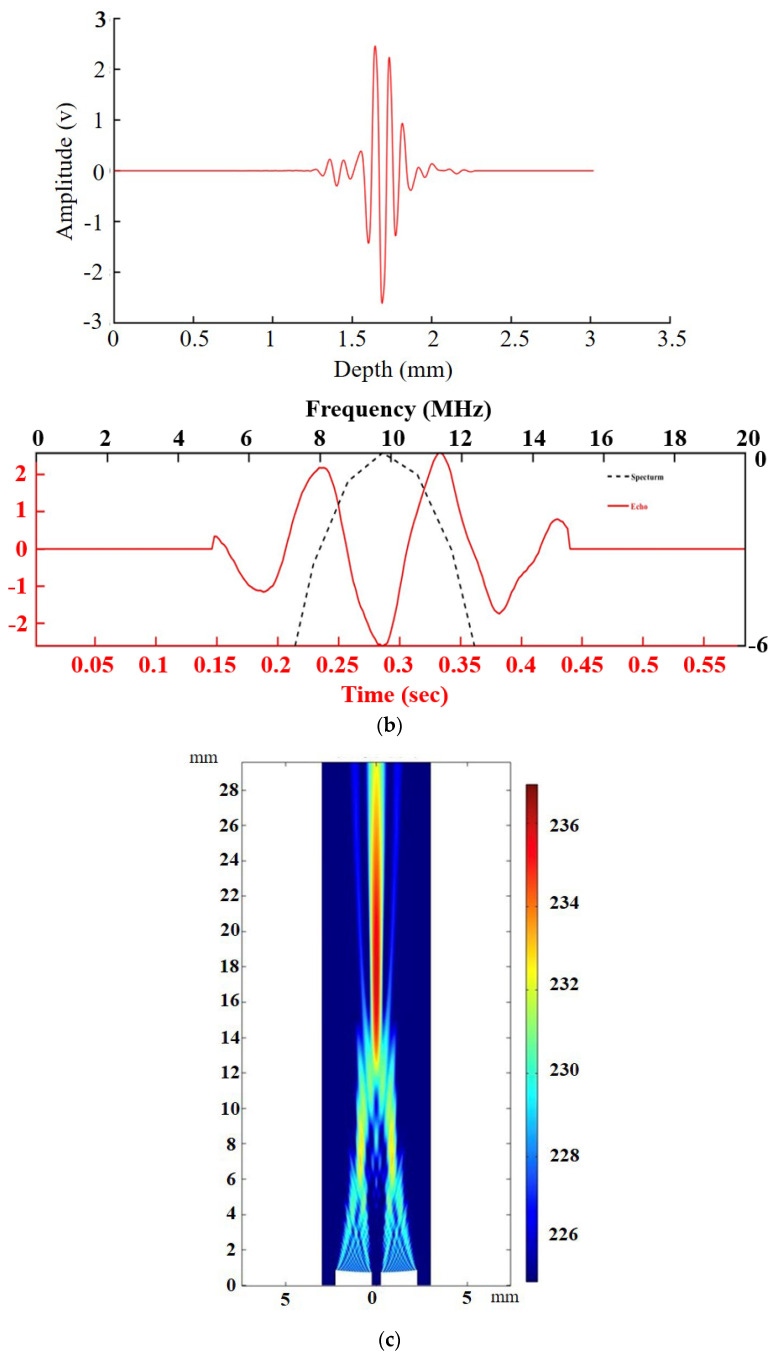
(**a**) Custom-made compressed focus dual−element ultrasound transducer and (**b**) pulse−echo ultrasound amplitude of the dual−element transducer signal, bandwidth 51%, and (**c**) the sound field simulation by COMSOL.

We developed a custom-made dual-element PMN-PT ultrasound transducer with a center frequency of 10 MHz for ultrasound transmission and reception. The PMN-PT has an excellent piezoelectric effect. The PMN-PT used in this study has a d33 value of 1620 pC/N, which is significantly higher than the d33 value of PZT, which is around 500 pC/N, can convert electrical and mechanical energy to each other to achieve the best effect of transmitting ultrasonic waves. The center frequency of the transducer was designed to match the image resolution and penetration depth requirements. Each PMN-PT transmitting and receiving element was cut into a 2 mm × 5 mm crystal pillar using a CNC (MDX-50, Roland, DG, Hamamatsu-shi, Japan) milling machine. Then, a microscope was used to inspect any damage, detachment, or mismatches in the shape and size of the matching layer and PMN-PT during the cutting process. The matching and backing layers were made on the front and back of the PMN-PT to increase the transmitting efficiency of ultrasonic waves, and to prevent the ultrasound waves from being reflected owing to the excessive acoustic impedance with the tissue. The backing layer was designed to prevent the PMN-PT from oscillating excessively and to lower the resolution. A steel ball with a diameter of 40 mm was used to press the probe’s focus, reaching a focal length of 20 mm and shaping it in an environment of 80 °C. Au sputtering was then conducted to connect the front matching layer with the brass tube. Finally, a 16 μm biocompatible parylene layer was coated on the Au sputtering layer to protect the probe and complete the production of the dual-element ultrasound transducer, as shown in Figure 1a.

The time-domain pulse-echo waveform and frequency-domain spectrum of the dual-element transducer measured at the focal position (approximately 20 mm) are shown in Figure 1b. The center frequency of the dual-element transducer was 9.7 MHz with a −6 dB bandwidth of 51%. A dual-element transducer sound field simulation using COMSOL Multiphysics (version 6.1, COMSOL Inc., Stockholm, Sweden) was performed (Figure 1c).

### 2.2. Optical Tracker

**Figure 2:**
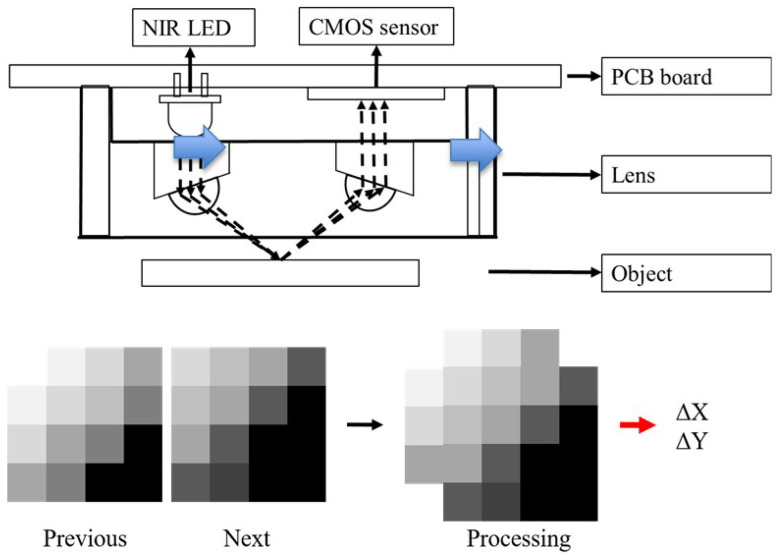
Schematic diagrams of optical tracker.

The optical tracker consisted of a CMOS sensor (ADNS-9500), infrared (IR) LED (842 nm), and digital signal processor (DSP). The CMOS sensor contained 30 pixels × 30 pixels. The pixel size was 10 µm × 10 µm. The resolution of optical tracking was 31 microns, which is about three times more precise than the resolution of magnetic tracking at approximately 100 microns, enabling more accurate tracking of displacement. The IR LED was incident on the skin surface at 30°, and a CMOS sensor captured 2D scattering images of the skin surface. With a high frame rate of the CMOS sensor, the digital signal processor can mathematically process the images using a cross-correlation function to obtain the displacement of the optical tracker. The two-dimensional (2D) displacement was measured using a Cartesian coordinate system. The displacement in each direction converges into the final displacement with the Pythagoras theorem. Subsequently, the DSP transmits the final displacement via Bluetooth to a laptop (Figure 2).

### 2.3. Measurement System

**Figure 3:**
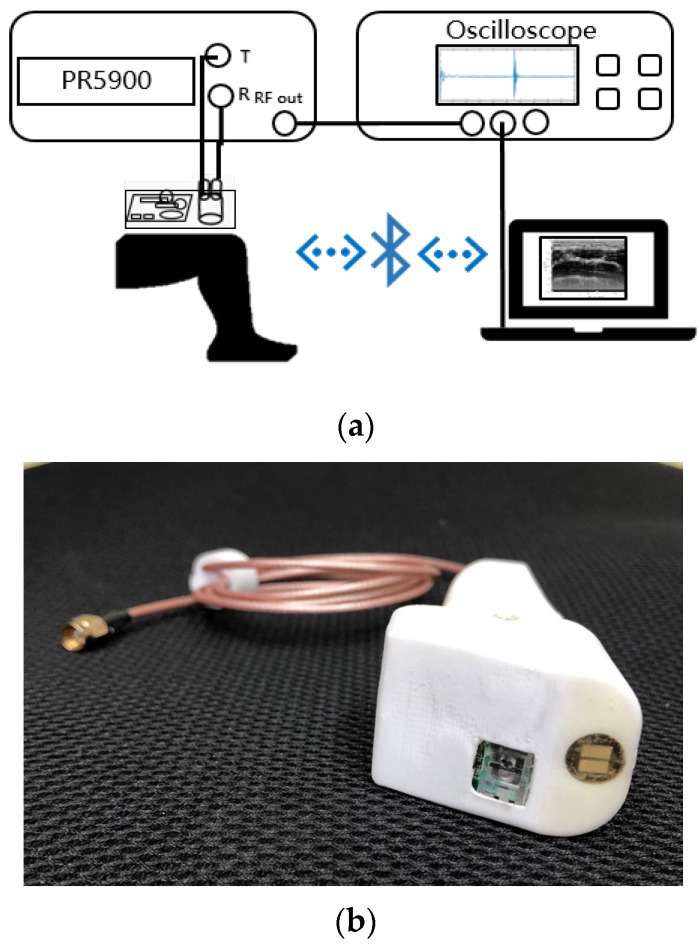
(**a**) Measurement system architecture diagram, and (**b**) photographs of the custom-made freehand scanner.

Figure 3a shows the entire measurement system consisting of a pulser/receiver (Panametrics 5900, Olympus Corporation, Tokyo, Japan), oscilloscope (Hero 64 Zi Lecroy, Chestnut Ridge, NY, USA), laptop, and customized dual-element ultrasonic probe (Figure 3b). The focal length of the probe was designed to be 20 mm, which is suitable for rectus femoris and arm muscle scans. The pulser receiver was operated at a pulse repetition frequency of 200 Hz. Thereafter, the reflected waveform was collected by the oscilloscope using 20 MHz sampling frequency, and was recorded and transferred to a laptop.

### 2.4. Image Processing

Using the recorded radio frequency (RF) ultrasound signal and the displacement information from the optical tracker, we combined these two data to build a real-time B-mode image. The RF signals were amplified by time-gain compensation, and then processed by log-compression and enveloped detection. The B-mode images were presented in 256 gray levels and integrated using LABVIEW 2018 (National Instruments, Austin, TX, USA). LabVIEW has both high-level and low-level language characteristics and contains numerous databases specifically created for DAQ, GPIB, and instrument control applications. Therefore, it is widely used in the industry to develop control programs.

### 2.5. Ultrasound Scanning

The participants were asked to sit in a neutral sitting position to relax their body. The measurement points were marked with black ink. Vaseline was smeared on the skin to eliminate the friction force that can cause errors during scanning.

**Figure 4:**
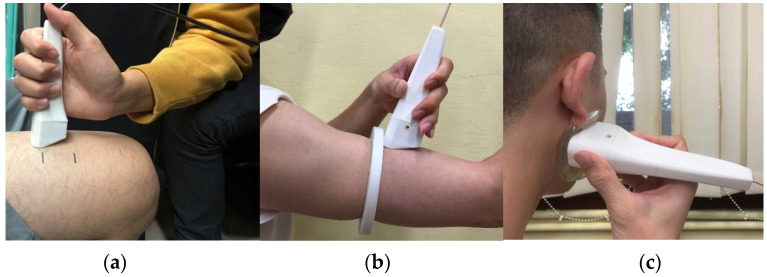
Ultrasound scanner measurement on subjects (**a**) thigh, (**b**) upper arm, and (**c**) cheek.

In this study, the target measurements were performed on three parts of the human body, namely, the rectus femoris of the thigh, upper arm muscle, and cheek muscle, as shown in Figure 4. The ultrasound transducer was scanned perpendicular to the long axis of the thigh to obtain a cross-sectional view of the rectus femoris. Three cross-sectional areas (CSAs) were scanned at 4 cm intervals from the knee tendon and rectus femoris junction towards the proximal end, 4 cm. In the cross-sectional area (CSA) image, the transverse thigh scan length was 15 cm, and a longitudinal scan was conducted 15 cm from the junction between the knee tendon and rectus femoris towards the proximal end. For the upper arm scan, we customized a magnetic mold to cover the mid-section of the upper arm to become a circular shape, and then the arm was directly above zero degrees in a clockwise direction for a 360° circular scan. To measure the masseter muscles of the cheeks, we placed a gel pad with a thickness of 1 cm on the cheek during the scan to clearly measure the superficial muscular aponeurotic system (SMAS) layer, starting from the top of the mandible and scanning down the masseter muscles to a total of 5 cm.

### 2.6. Comparison of Ultrasound Transducer

In this study, three types of transducers were used to verify the image quality of the dual-element ultrasound scanner with miniature optical position tracker. These included a custom-made single-element ultrasound transducer made of PMN-PT material, a frequency of 10 MHz, a compression focus depth of 2 cm, and a chip size of 2 mm × 5 mm; a custom-made dual-element ultrasound transducer made of PMN-PT material, a frequency of 10 MHz, a compression focus depth of 2 cm, and a chip size of 2 mm × 5 mm; and a commercially available handheld wireless array ultrasound transducer (LU700L, LELTEK Inc., Taiwan, China) with a frequency range of 5–10 MHz, a maximum scanning depth of 6 cm, and a probe face diameter of 5 cm for comparison. We measured the lateral and axial resolutions of the transducers by employing a 0.1 mm steel wire phantom. Our findings indicated that the single-element transducer achieved a lateral resolution of 0.4 mm and an axial resolution of 0.1 mm. In contrast, the dual-element transducer exhibited a lateral resolution of 0.3 mm and an axial resolution of 0.1 mm. Additionally, our assessment revealed that the handheld wireless array ultrasound transducer had a lateral resolution of 0.2 mm and an axial resolution of 0.2 mm. To ensure consistency in human body scanning comparisons, the scanning process was standardized with a center frequency of 10 MHz and a gain value of 20 dB at a scanning depth of 3.5 cm. A mold was fixed in the target scanning area on the right thigh to ensure consistent scanning path, and a large amount of conductive gel was applied to the scanning area to avoid inconsistent imaging. The three probes were sequentially scanned within the same period to ensure any physiological or environmental changes did not affect the results. The three probes were sequentially scanned at the same part and the same time to ensure that any physiological or environmental changes did not affect the results.

## 3. Results

**Figure 5:**
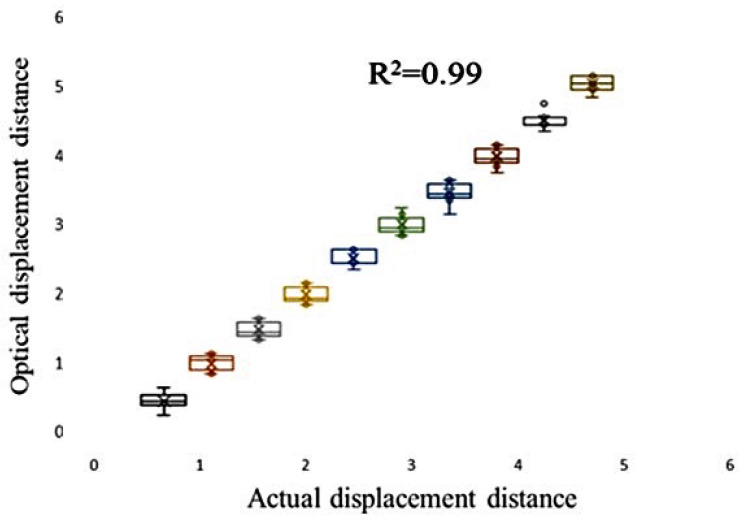
Statistical results of the optical displacement distance repeatability test.

To verify the accuracy of the optical displacement distance, we placed a 5 cm mold on the skin to verify the optical tracker movement with a fixed distance and range. Thereafter, we recorded the calculated displacement distance and actual displacement distance at each 5 mm movement, up to a total distance of 5 cm. The optical tracker performed a total of ten movements in the mold, and the measured results between the actual and measured values are shown in Figure 5. The mean of ten simple linear regressions (R^2^ = 0.99), *p*-value < 0.05, and the standard deviation was 0.12 mm.

**Figure 6:**
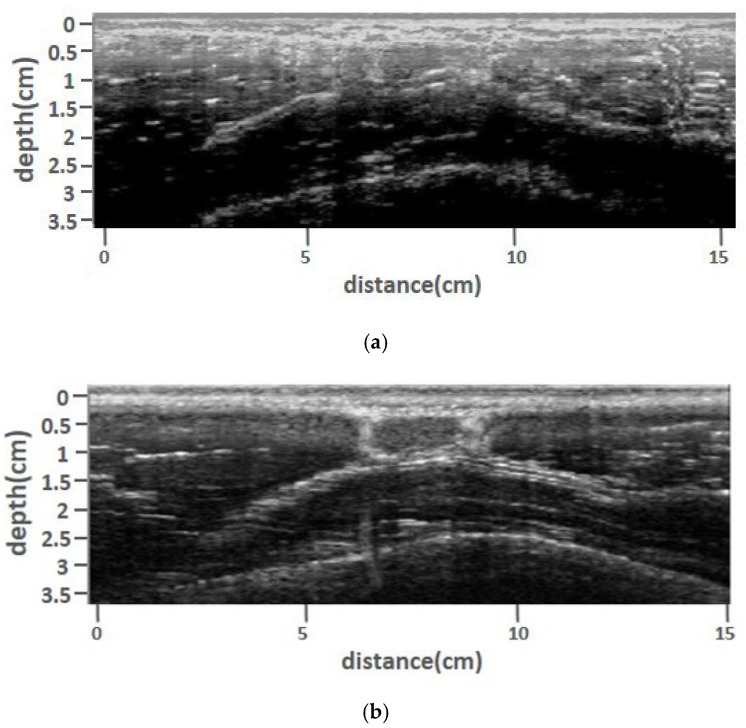

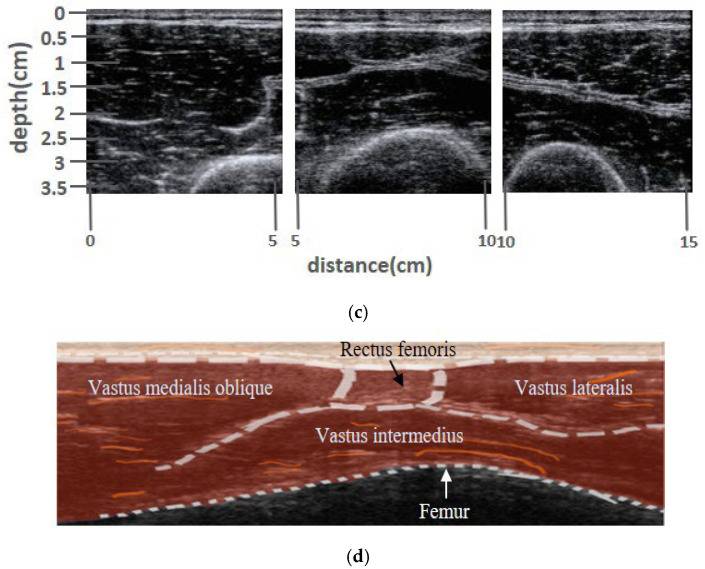
Rectus femoris ultrasound image scanning by (**a**) single-element transducer, (**b**) dual-element transducer, (**c**) LELTEK handheld wireless array ultrasound transducer, and (**d**) the anatomy schematic of the rectus femoris.

We measured and compared the quadriceps B-mode image measured using a single-element, dual-element transducer, and LELTEK handheld wireless array ultrasound transducer. Figure 6a–d shows that the upper border of the rectus femoris muscle was approximately 5–10 mm below the skin. The bright white lines within 5 mm of depth are subcutaneous tissue, followed by the rectus femoris muscle at a depth of 5–12 mm. We can observe the outline of the muscle, while the vastus medialis and vastus lateralis are adjacent to the rectus femoris. Furthermore, the larger muscle is the vastus intermedius, and the texture of the myofascia and other detailed tissues can be observed in the image. At 30 mm, there was a strong arc-shaped reflection from the femur.

**Figure 7:**
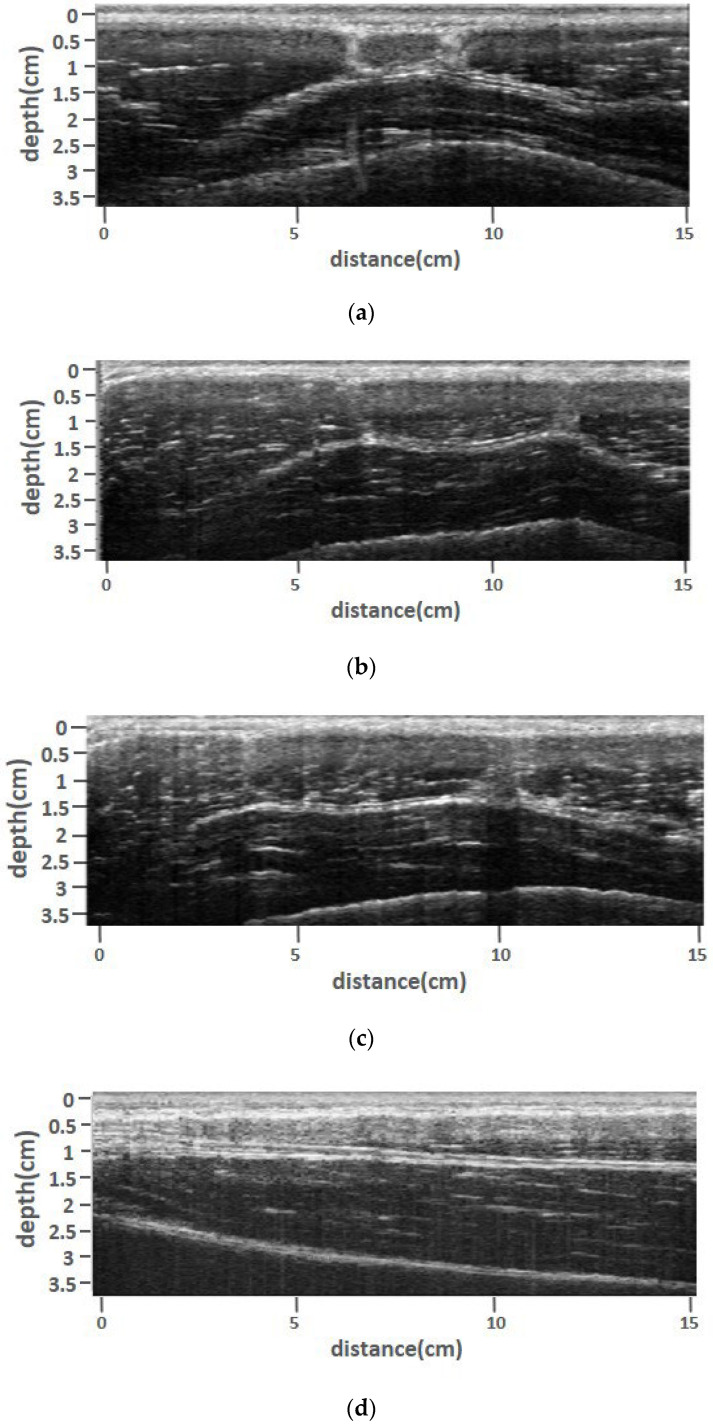

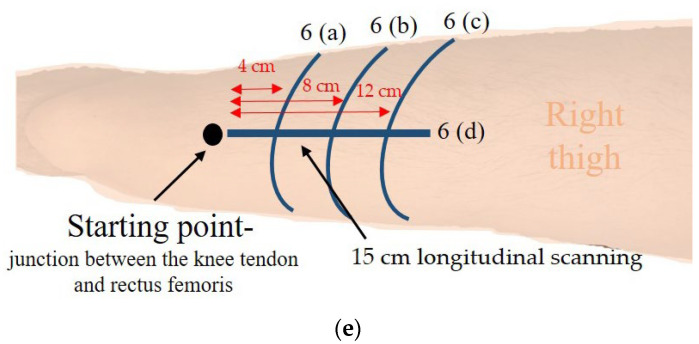
Different sections of rectus femoris image scanning by ultrasound scanner: (**a**) 4 cm intervals from the starting point towards the proximal end; (**b**) 8 cm intervals from the starting point towards the proximal end; (**c**) 12 cm intervals from the starting point towards the proximal end; (**d**) 15 cm longitudinal scanning of rectus femoris. (**e**) The schematic of the longitudinal and transverse scanning paths.

The starting point of the ultrasound rectus femoris measurement was the junction between the knee tendon and rectus femoris. Figure 7a shows scanning at 4 cm intervals from the starting point toward the proximal end, targeting the rectus femoris muscle at a depth within the range of 5–12 mm. Furthermore, Figure 7b shows scanning at 8 cm intervals from the starting point toward the proximal end, targeting the rectus femoris muscle at a depth within the range of 5–15 mm. Figure 7c depicts scanning at 12 cm intervals from the starting point toward the proximal end, targeting the rectus femoris muscle at a depth within the range of 5–17 mm. As the scan moves towards the middle of the thigh, the cross-sectional area of the rectus femoris muscle becomes larger, while a clear and complete muscle boundary can still be observed. Figure 7d shows the longitudinal scanning image from the top of the image to the bottom. We can observe subcutaneous adipose tissue from the end of the tendon to the middle of the thigh, the upper and lower borders of the rectus femoris, and the femur. Furthermore, we can accurately measure subcutaneous fat thickness (0–5 mm), rectus femoris thickness (5–12 mm), and bone depth (22–35 mm) under the scanning path, as shown in Figure 7d).

**Figure 8:**
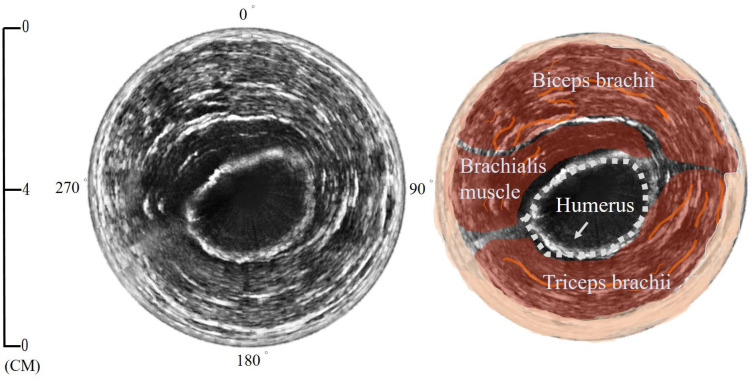
Upper arm anatomy schematic and circular image scanning by ultrasound scanner.

The upper arm circular image scan is shown in Figure 8. A complete circle of subcutaneous adipose tissue (4 mm) is observed in the image. At 1–2 cm (270°–90°), we can observe the muscle outline of the biceps, myofascia, and other detailed tissue textures. At 3–4 cm, the humerus and brachialis muscles can be observed. At 0–4 cm (90°–230°), we can observe the muscle outline of the triceps brachii and the detailed tissue texture of the myofascial.

**Figure 9:**
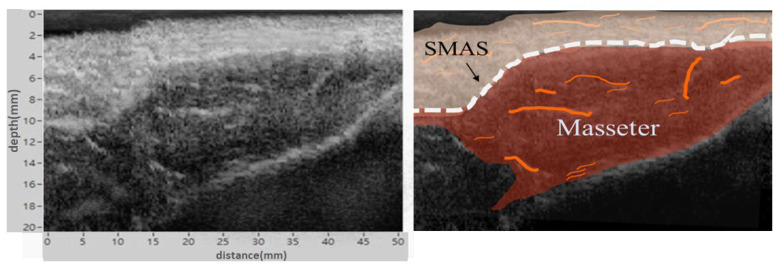
Cheek anatomy schematic and masseter, SMAS layer images scanning by ultrasound scanner.

Figure 9 shows the cheek scan, which was conducted with a 1 cm layer of gel pad on the cheek. We can observe that the SMAS layer is located approximately 4 mm subcutaneously and the masticatory muscle is located 5–20 mm beneath the skin. The contour of the muscle, myofascial texture, and fine texture of the tissue can easily be observed with the naked eye.

## 4. Discussion

In this study, we developed a handheld and user-friendly single-channel dual-element ultrasound scanner with a miniaturized optical positioning tracker to obtain displacement information during ultrasound scanning and imaging. Optical tracking technology enables the use of a custom single-channel dual-element ultrasound scanner to obtain wide B-mode longitudinal and transverse cross-sectional images, as well as circular cross-sectional images, through a single, continuous scan of the skin using a handheld scanner.

We identified several advantages of using the optical positioning tracker. These included compactness in size, low cost, fast response, high positioning resolution, and reliable directional measurement consistency within the test volume [30]. As shown in Figure 5, we demonstrate the accuracy of the optical positioning tracker through repeatability tests. The results show a high R^2^ value of 0.99, low *p*-value of less than 0.05, and small standard deviation of 0.12 mm. These findings demonstrate the effectiveness of the optical positioning tracker for recording the ultrasound scanner. In addition, we observed that optical tracking and electromagnetic positioning methods are widely used in ultrasound imaging. Lang et al. reported that an optical tracking method offers superior positioning accuracy and is free from metal distortion [17]. Peters et al. investigated various trackers and concluded that optical tracking is accurate and reliable [31]. This result is consistent with our findings.

Furthermore, we compared the performance of single-element and dual-element transducers, as shown in Figure 6. We explored the optimal design parameters of a dual-element transducer, which consisted of two rectangular 2 × 5 mm elements with a symmetrical or angled arrangement. Figure 6a,b demonstrate that images produced by the dual-element transducer exhibit improved contrast and surpass those produced by the single-element transducer in terms of muscle boundary and femur outline visibility at a depth of 30 mm. The single-element transducer, due to its chip being perpendicular to the test tissue, can cause multiple reflections between the transmitted and received signals, resulting in the reverberation artifact. Furthermore, using the same chip for both transmitting and receiving signals can lead to the near-field noise. This making it difficult to identify the muscle boundaries of the rectus femoris, ultimately decreasing the accuracy of muscle thickness and area calculations. In contrast, the images produced by the dual-element transducer exhibited uniform overall intensity and contrast, significantly reducing image noise. The outline of the rectus femoris muscle on both sides was particularly noticeable. The single-element transducer attempted to produce reflected echoes when the boundaries of the rectus femoris muscle were parallel to the chip direction. Conversely, the dual-element transducer effectively addressed this issue by adopting an angled setting between the two chips, rendering it ideal for muscle scanning. Several studies have demonstrated the superiority of the dual-element transducer design. Zhao et al. showed that dual-element transducers can achieve a higher near-surface resolution and signal-to-noise ratio than single-element transducers [32]. In Figure 6b,c, the muscle image quality obtained by the dual-element ultrasound scanner with a miniature optical position tracker is similar to that of handheld array probes currently available on the market, and it can present clear muscle contours. Moreover, the scanner developed in this study can acquire a 15 cm scan image in a single scan, while the array probe used for comparison requires three separate scans due to its limited scan range of 5 cm in diameter. After accessing and stitching the images together, there may be issues with mismatched bone curvature, and the lack of a positioning system may result in images that are not to scale.

A custom-made handheld ultrasound scanner with optical positioning tracker can provide seamless scanning over a large area without any limitations on the scanning length, as shown in Figure 7 for a 15 cm or even longer scanning length. During the scanning of the middle portion of the thigh, the cross-sectional area of the rectus femoris muscle can still be clearly and comprehensively observed, although it is sufficiently large and wide. Conventional array probes cannot capture the complete cross-sectional view of muscles because of limitations in the probe. Although high-end array ultrasound machines equipped with image stitching technology can achieve this, they are expensive. In addition to cross-sectional muscular measurements, the entire muscle can be scanned longitudinally to obtain complete muscle thickness values for predicting muscle volume or mass, as shown in Figure 7d. This handheld device is a user-friendly tool for simple muscle volume assessments. To accurately calculate the cross-sectional area of the muscle in clinical settings, a noticeable muscle boundary, which can be obtained by encircling the region of interest, should be obtained. This innovative device provides a low-cost and convenient method of acquiring and calculating cross-sectional images of the muscle from one continuous scan. Moreover, it is not limited by the scanning length.

We investigated the use of the custom-made ultrasound scanner for 360° scanning, as shown in Figure 8. This device provides an easy procedure for obtaining circular cross-sectional images of large muscle areas similar to CT scans. This innovative approach to ultrasound imaging can improve diagnoses and render low-cost and portable point-of-care ultrasound equipment available. Furthermore, this custom-made handheld ultrasound scanner may provide medical imaging and services to remote and underserved communities.

In this study, we also evaluated the use of the custom-made ultrasound scanner for imaging shallow fascia and muscle tissues by placing a 1 cm thick layer of an ultrasound gel pad on the cheek, as shown in Figure 9. The gel pad serves as a cushion, enabling the probe to focus near the center between the SMAS layer and the lower boundary of the masseter muscle. The images produced show that the muscle outline, fascia texture, and finer tissue texture can easily be differentiated with the naked eye. This system provides a cost-effective and easily maneuverable imaging solution for extensive investigations of shallow tissues.

Notably, certain guidelines are required to achieve optimal results with the ultrasound scanner. To reduce friction and ensure a smooth movement, an even layer of lotion or petroleum jelly should be applied to the scanning area. Furthermore, moderate contact pressure and an ultrasound gel should be applied between the skin and the probe to ensure good acoustic contact and prevent image errors caused by air. The movement of the scanner should be maintained at a constant speed of less than 5 cm/s for image consistency. By following these guidelines and with some practice, the operator can easily handle the handheld ultrasound device and obtain desirable imaging outcomes.

## 5. Conclusions

In this study, we developed a 10 MHz PMN-PT dual-element ultrasound transducer to improve the resolution of near-field signals and effectively reduce noise, and then combined it with an optical position tracker. Using this innovative technique, we significantly improved the outcomes compared to the single element in the muscle boundary and the outline of the femur, and it was not limited to the scanning length. Moreover, the custom dual-element transducer equipped with an optical tracker boasts high maneuverability and a low cost, and lacks a scanning range limitation.

## Data Availability

Not applicable.
